# Supplementary material for: Spatiotemporal Transformer Neural Network for Time-Series Forecasting
Source: Entropy (Basel). 2022 Nov 14;24(11):1651. doi: 10.3390/e24111651 (PMC9689721; doi:10.3390/e24111651)
Supplement: Supplementary file 1 [file entropy-24-01651-s001.zip › entropy-1878274-supplementary.pdf]

SUPPLEMENTAL INFORMATION FOR

Spatiotemporal transformer neural network (STNN) for multivariable short-term time-series prediction

content

1. Supplementary related works 2
    1.1 Delay-embedding for spatial-temporal transformation equation 2
    1.2 Transformer neural network for time series prediction 3
2. Supplementary Baselines 4
3. Supplementary Datasets 5
    3.1 Nonlinear Pendulum 5
    3.2 Lorenz system 5
    3.3 Gene expression 6
    3.4 Traffic speed (TS) 6
    3.5 Solar irradiance 6
    3.6 Traffic Flow (TF) 6
4. Supplementary Experiments 8
    4.1 Nonlinear Pendulum 8
    4.2 Lorenz system 8
    4.3 Gene expression 10
    4.4 TS 10
    4.5 Solar irradiance 10
    4.6 TF 11
    4.7 Robustness 11
    4.8 Dimension experiment 12
    4.9 The perfomance of STNN 20
5. Supplementary Figure 23
    Supplementary Figure 5.1 The workflow of each computational experiment 23
6. Supplementary Algorithm 24
    Supplementary Algorithm 1 The algorithm of each computational experiment 24
7. Supplementary codes 25

1. Supplementary related works

1.1 Delay-embedding for spatial-temporal transformation equation

For a general discrete-time dynamical system, Eq. 1.1 defines the dynamical evolution of its state.

$$X^{t+1} = \phi(X^t) \quad (1.1)$$

Here, $X^t = (x_1^t, x_2^t, \dots, x_D^t)'$ are defined on a D-dimensional space at time step t, where symbol $'$ means the transpose of a vector. The map $\phi: \mathbb{R}^D \to \mathbb{R}^D$ is a nonlinear function, which pushes states from time t to time $t+1$.

To bridge the spatial information and the temporal evolution information, we let $\mathbf{Y}^t = (y^t, y^{t+1}, \dots, y^{t+L-1})' = (x_{target}^t, x_{target}^{t+1}, \dots, x_{target}^{t+L-1})'$ which are the values of one target variable selected from X for (L-1)-step-ahead prediction with L>1. Note $\mathbf{X}^t$ is spatial/high-dimensional information due to multiple (D) variables at one time point t while $\mathbf{Y}^t$ is temporal information due to one variable at multiple (L) time points. When the system of Eq.(2.1) is on a steady state or on a manifold $\mathcal{V}$ with dimension *d*, based on the Takens' embedding theorem, we can construct the following spatiotemporal information (STI) transformation equation, which maps the D-dimensional data $\mathbf{X}^t$ to L-dimensional data $\mathbf{Y}^t$.

$$\Phi(\mathbf{X}^t) = \mathbf{Y}^t = (y^t, y^{t+1}, \dots, y^{t+L-1}) \quad (1.2)$$

where generally, D>>L and L>2d. Clearly, the spatiotemporal information (STI) transformation equation transforms the available/previous spatial information $\mathbf{X}^t$ of multiple variables to the future temporal information $\mathbf{Y}^t$ of one target variable at each time point t. If we measure a time-series with M points, we can rewrite Eq.1.2 in a matrix form of Eq.1.3.

$$\begin{bmatrix} \Phi_1(X^1) & \Phi_1(X^2) & \dots & \Phi_1(X^M) \\ \Phi_2(X^1) & \Phi_2(X^2) & \cdots & \Phi_2(X^M) \\ \vdots & \vdots & \ddots & \vdots \\ \Phi_L(X^1) & \Phi_L(X^2) & \cdots & \Phi_L(X^M) \end{bmatrix} = \begin{bmatrix} y^1 & y^2 & \cdots & y^M \\ y^2 & y^3 & \cdots & \hat{y}^{M+1} \\ \vdots & \vdots & \ddots & \vdots \\ y^L & y^{L+1} & \cdots & \hat{y}^{M+L-1} \end{bmatrix} \quad (1.3)$$

Since the observation variables are up to time step M, the ^ indicates that the values of target variable y from time steps M+1 to M+L-1 need to be predicted in addition to the maps Φ_i for $i = 1, \dots, L$, given X^t for t=1, …, M. Thus, we can have (L-1)-step-ahead prediction of a target variable y by solving Φ_i and $\mathbf{Y}^t$ of Eqn.2.3 provided that X^t for t=1, …, M are available. Generally, even if the dimension D of the original system is very high, the dimension d of its steady state or manifold is very low for most of real-world systems, i.e. D>>d. Thus, we generally choose a small d by letting L=2d+1 in the computation of Eq.1.3.

Several works have tried to predict high-dimensional short-term time series with STI equation. For example, Ma et al., firstly explored STI transformation equation and established a framework, named randomly distributed embedding (RDE), for one-step-ahead prediction of short-term time series. The novelty of this RDE framework roots in exploiting the information embedded in many low-dimensional non-delay attractors as well as in an appropriate use of the distribution of the target variable for prediction. Chen et al., developed an auto-reservoir computing framework, named Auto-Reservoir Neural Network (ARNN), to approximate the nonlinear STI equation to a linear-like form, which can efficiently make multi-step-ahead prediction based on a short-term high-dimensional time series. Such ARNN transformation equivalently expands the sample size but its linear-like approximation sacrifices the accuracy to some extent although it has great potential in practical applications of artificial intelligence and machine learning

1.2 Transformer neural network for time series prediction

Transformer has been widely used in the field of natural language processing, which is described in details by Vaswani et al.. Unlike sequence-aligned models, Transformer processes entire sequence of data and leverages classical self-attention mechanism to capture global dependencies of the sequence $\mathbf{X}$ as Eq. 2.3, i.e..

$$\mathrm{Attention}(Q,K,V) = \mathrm{Softmax}(\frac{QK^T}{\sqrt{d_k}} \cdot \mathrm{Mask})V \quad (2.3)$$

where query matrix $Q = XW_Q$, key matrix $K = XW_K$ and value matrix $V = XW_V$ are transformed by X respectively. W_Q, W_K and W_V are learnable parameter matrices. Note that a mask matrix is applied to filter out rightward attention by setting all upper triangular elements to $-\infty$, in order to avoid future information leakage.

However, at present, the transformer structure has not been well studied for processing high-dimensional short-term time series data. Besides, only a few studies consider the effective modeling of time series from the perspective of attention mechanism. For example, Shih et al., propose an attention mechanism to extract temporal patterns, which successfully captures the temporal information of time series. Moreover, self-attention enables Transformer to focus on different aspects of temporal patterns, and thus has a potential for time series prediction by reducing the unrelated variables.

2. Supplementary Baselines

Baselines: We select six time-series forecasting methods, including autoregressive integrated moving average (ARIMA), support vector regression with linear kernel (SVR), support vector regression with radial basis function (RBF), recurrent neural network (RNN), and Koopman autoencoder (KAE). To better explore the performance of continuous attention mechanism, we incorporate the canonical attention mechanism as STNN*.

Metrics: We use Pearson Correlation Coefficient (PCC) and normalized Root mean square error (RMSE) to evaluate the difference between the prediction and the real results.

$$\mathrm{PCC} = \frac{\sum_{i=m}^{m+L-1}(\hat{y}^i - \hat{\mu})(\mathrm{y}^i - \mu)}{\hat{\sigma}\sigma} \quad (2.1)$$

$$\mathrm{nRMSE} = \frac{\sqrt[2]{\frac{1}{L}\sum_{i=m}^{m+L-1}\|\hat{y}^i - y^i\|^2}}{\sigma} \quad (2.2)$$

Here, $\hat{y}^i$ is the predicted value at the time step i. $\hat{\mu}$ and μ is the mean value of the predictive and true data, respectively. $\hat{\sigma}$ and σ is the variance of the predicted data and true data, respectively.

3. Supplementary Datasets

The datasets for STNN is available at https://github.com/347251369/STNN

3.1 Nonlinear Pendulum

The nonlinear pendulum is a classic textbook example for dynamical systems, which is used for benchmarking models. We generated a dataset of nonlinear pendulum with 80 data points. This problem can be modeled as a second order ODE by：

$$\frac{d^2\theta}{dt^2}+\frac{g}{l}sin\theta=0 \tag{3.1}$$

where the angular displacement from an equilibrium is denoted by $\theta\in[0,2\pi)$. We use l and g to denote the length and gravity, respectively, with $l=1$ and $g=9.8$ in practice. We consider the following initial conditions $\theta(0)=\theta_0$ and $\theta^{\cdot}(0)=0$. The motion of the pendulum is approximately harmonic for a small amplitude of the oscillation $\theta_0\ll 1$. However, the problem becomes inherently nonlinear for large amplitudes of the oscillations. Through Eq.3.1 we get the 2-dimention position time-series $\{x^t\}$.

In addition, we map the sequence $\{x^t\}$ to a high-dimensional space via a random orthogonal transformation to obtain the training snapshots, i.e., $\mathbf{P}\in R^{64\times 2}$ such that $f(t) = PX^t$ for any t. Finally, we get the 64-dimension nonlinear pendulum data.

In robustness experiment, we employ a white Gaussian noise term to the 64-dimension nonlinear pendulum data by Eq.3.2:

$$\{\tilde{x}_i^t=x_i^t+\varepsilon_i \tag{3.2}$$

Where $\tilde{x}$ represent the i-th dimension time-series data with noise at time step t.. And ε_i represent the noise terms for $\tilde{x}_i^t$, which are modeled as stochastic variables sampled from normal distributions $\mathcal{N}(0,\sigma^2)$ with zero mean and standard deviation σ. The standard deviation σ can be used as the noise intensity.

3.2 Lorenz system

The 90-dimension coupled Lorenz system is a classical system for studying essential dynamical characteristics of nonlinear systems, which is of great significance to model high-

dimensional nonlinear natural systems. We use the 90-dimension coupled Lorenz system which is composed of 30 subsystems formed as follow:

$$\begin{cases} \dot{x}_i = \alpha(y_i - x_i) + cx_{i-1} \\ \dot{y}_i = \rho x_i - y_i - x_i z_i \\ \dot{z}_i = x_i y_i - \beta z_i \end{cases} \tag{3.3}$$

where $i \in [1,30]$ denotes the ith subsystem, and c is the coupled factor for coupling the ith subsystem with the previous subsystem. This experiment sets $c = 0.1, \rho = 28, \beta = 8/3, \alpha = 10$ as the constants for each subsystem. Since real world data exists a lot of noise, we employ a white Gaussian noise term to simulate the real situation:

$$\begin{cases} \tilde{x} = x + \varepsilon_x \\ \tilde{y} = y + \varepsilon_y \\ \tilde{z} = z + \varepsilon_z \end{cases} \tag{3.4}$$

where $\tilde{x}$, $\tilde{y}$ and $\tilde{z}$ represent data with noise. And ε_x, ε_y and ε_z represent the noise terms for x, y and z, which are modeled as stochastic variables sampled from normal distributions $\mathcal{N}(0, \sigma^2)$ with zero mean and standard deviation σ. The standard deviation σ can be used as the noise intensity.

3.3 Gene expression

We use the gene expression data of circadian rhythm in rat to test the STNN. Because of the complicated gene regulation mechanism, a large number of genes in a living organism usually form a high-dimensional dynamical system to facilitate various biological functions. Here, we employ STNN to predict the expression of a number of genes for circadian rhythm, which are measured by Affymetrix microarray on the laboratory rat with 84 genes and 22 time steps. Each time step is 2 hours.

3.4 Traffic speed (TS)

Traffic speed prediction is a vital topic in industry, which helps drivers make better travel decisions, alleviate traffic congestion and reduce carbon emissions. Here, we employ STNN to predict the traffic flow based on a dataset collected from 207 loop detectors at Highway 134 of Los Angeles County.

3.5 Solar irradiance

Prediction of solar irradiance is essential to minimize the energy costs and provide high power quality. Here, we employ STNN to predict the solar irradiance based on a dataset generated from 51 sampling sites in Wakkanai, Japan. The 51 sampling sites have formed a system to reflect the changes of solar irradiance by making a record every 10 minutes since 2011.

3.6 Traffic Flow (TF)

The data is originally collected from California department of transportation and describes the road occupy rate of Los Angeles County highway network. We here use the subset in which selects 228 sensors randomly with 40 days data points for each sensors.

4. Supplementary Experiments

4.1 Nonlinear Pendulum

We generate 80 data points by Eq.3.1. We divide the gene expression time series into a training and testing datasets. The training dataset is comprised of the first 63 steps, and the rest 17 steps is for the testing dataset. The results are list in Table S1.

Table S1. The prediction result of pendulum data.

Dataset	Metric	Methods						
		STNN	STNN*	ARIMA	SVR	RBF	RNN	KAE
y_1	PCC	**0.998**	0.958	0.097	0.983	0.992	0.901	0.975
	nRMSE	**0.053**	0.420	0.845	0.106	0.084	0.268	0.132
y_2	PCC	0.991	0.978	0.970	**0.999**	0.993	0.946	0.995
	nRMSE	0.237	0.449	0.317	0.343	0.390	0.268	**0.135**
y_3	PCC	0.998	0.948	0.712	0.992	0.995	0.969	**0.999**
	nRMSE	**0.118**	0.660	0.666	0.166	0.173	0.271	0.125
y_4	PCC	0.990	0.654	-0.293	0.990	**0.994**	0.972	0.993
	nRMSE	0.174	0.829	0.889	0.096	**0.113**	0.226	0.126

4.2 Lorenz system

We generate 80 data points by Eq.3.2. We divide the gene expression time series into a training and testing datasets. The training dataset is comprised of the first 61 steps, and the rest 19 steps is for the testing dataset. The results are list in Table S2. In the experiment, Gaussian noise is set $\sigma = 0$.

Table S2. The prediction result of lorenz data.

Dataset	Metric	Methods						
		STNN	STNN*	ARIMA	SVR	RBF	RNN	KAE
y_1	PCC	**0.998**	-0.926	0.982	-0.970	-0.998	0.951	-0.691
	nRMSE	**0.070**	3.770	0.306	2.000	1.905	2.300	3.088
y_2	PCC	**0.985**	0.085	0.717	-0.271	-0.702	0.417	-0.733
	nRMSE	**0.157**	1.278	0.658	1.641	1.797	1.942	2.834
y_3	PCC	**0.998**	-0.377	0.929	0.988	0.881	0.325	0.310
	nRMSE	**0.126**	2.407	1.071	0.676	0.978	1.123	1.142
y_4	PCC	**1.000**	-1.000	0.998	-0.972	-0.967	-0.461	-0.985
	nRMSE	**0.035**	2.350	0.447	2.005	1.722	1.899	3.453

In order to clearly present the experiment of Lorentz system, we detail the Lorenz system experiment as follows.

We generate a 90-dimensional time series $X^1, \ldots, X^{80} \in \mathbb{R}^{90}$ with 80 steps by Eq 3.1. Then, we divide the generated time series into a training and testing datasets. The training dataset is comprised of 61 initial steps, and the rest is for the testing dataset. After we employ the preset parameters $(D = 90, M = 61, L - 1 = 19)$ to predict the 19-step-ahead values for four different target variables $(y_1, y_2, y_3 \text{ and } y_4)$ in Fig. S4.1.

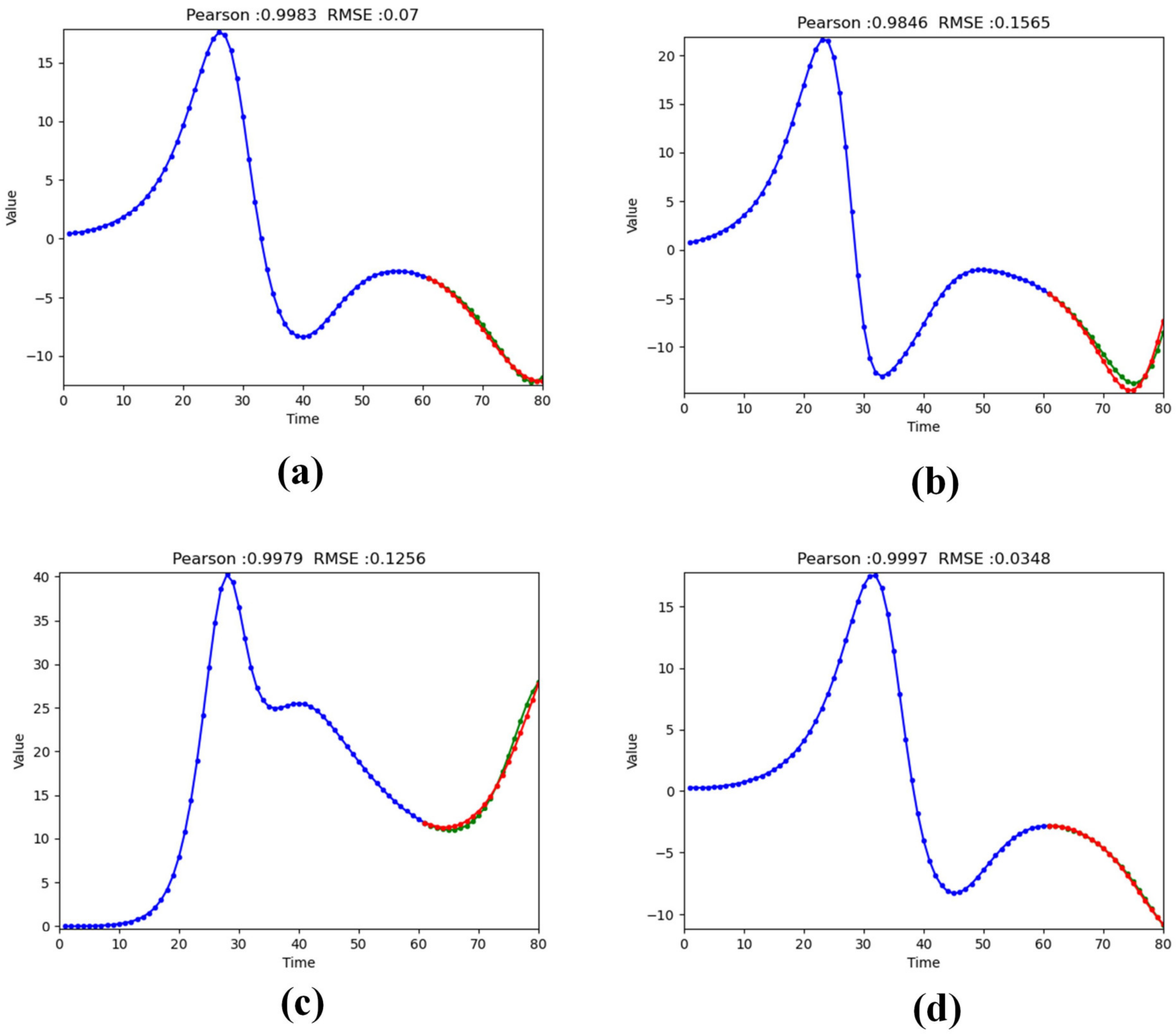

Figure S4.1 The prediction results of the Lorenz system by STNN. The abscissa represents the time range (80 steps), and the ordinate represents the target/prediction variable. The blue line represents the training data, the green line is the test data and the red line is the prediction data. (a-d) respectively represent the prediction results of four different target predicted variables of Lorentz subsystem.

4.3 Gene expression

We divide the gene expression time series into a training and testing datasets. The training dataset is comprised of the first 16 steps, and the rest 6 steps is for the testing dataset. We employed STNN to predict the 6-step-ahead values for four different target variables, which are shown in Table S3.

Table S3. The prediction result of gene data.

**Dataset**	**Metric**	**Methods**						
		STNN	STNN*	ARIMA	SVR	RBF	RNN	KAE
y_1	PCC	0.453	0.344	0.470	**0.504**	0.497	-0.117	0.446
	nRMSE	**0.671**	1.039	1.080	0.720	1.158	1.831	1.952
y_2	PCC	0.252	-0.151	0.067	0.118	**0.315**	-0.702	-0.032
	nRMSE	**0.540**	1.632	0.995	0.705	0.837	0.982	2.467
y_3	PCC	0.463	**0.577**	-0.309	0.150	0.358	0.832	-0.684
	nRMSE	0.707	0.691	1.114	1.074	1.260	**0.548**	1.107
y_4	PCC	0.412	0.754	0.745	**0.843**	0.615	0.673	0.012
	nRMSE	0.712	0.871	0.603	**0.550**	0.813	1.078	2.267

4.4 TS

We divide the traffic flow time series into a training and testing datasets. The training dataset is comprised of the first 71 steps, and the rest 19 is for testing dataset. We employed STNN to predict the 19-step-ahead values for four different target variables, which are shown in Table S4.

Table S4. The prediction result of TS data.

**Dataset**	**Metric**	**Methods**						
		STNN	STNN*	ARIMA	SVR	RBF	RNN	KAE
y_1	PCC	**0.948**	0.931	0.595	0.544	0.442	0.650	-0.730
	nRMSE	**0.354**	0.474	0.731	1.134	1.570	0.785	1.197
y_2	PCC	0.757	**0.760**	-0.396	0.296	0.655	0.105	0.401
	nRMSE	0.642	**0.639**	1.199	1.304	1.445	1.152	0.818
y_3	PCC	**0.917**	0.857	0.473	0.507	0.460	0.215	-0.317
	nRMSE	**0.544**	0.645	1.457	1.038	1.202	1.287	1.193
y_4	PCC	**0.842**	0.125	0.360	0.709	0.622	-0.178	-0.246
	nRMSE	**0.476**	1.262	0.941	1.426	0.996	1.702	1.894

4.5 Solar irradiance

We divide the solar irradiance time series into a training and testing datasets. The training dataset is comprised of the first 301 step solar irradianceps, and the rest 149 is for the testing

dataset. We employed STNN to predict the 149-step-ahead values for four different target variables, which are shown in Table S5.

Table S5. The prediction result of solar irradiance data.

Dataset	Metric	Methods						
		STNN	STNN*	ARIMA	SVR	RBF	RNN	KAE
y_1	PCC	0.913	**0.927**	-0.390	0.774	0.844	0.133	0.363
	nRMSE	0.497	**0.440**	1.221	0.760	0.999	1.982	2.030
y_2	PCC	0.945	**0.938**	0.379	0.847	0.802	0.054	-0.081
	nRMSE	0.450	**0.401**	1.053	0.546	0.828	1.247	1.164
y_3	PCC	**0.994**	0.994	0.394	0.745	0.852	0.231	-0.017
	nRMSE	**0.108**	0.110	1.178	0.725	1.028	1.335	1.538
y_4	PCC	**0.942**	0.945	0.370	0.207	0.824	0.202	-0.223
	nRMSE	0.432	**0.428**	1.064	1.205	0.881	1.755	1.676

4.6 TF

We divide the traffic speed time series into a training and testing datasets. The training dataset is comprised of the first 31 stesolar irradianceps, and the rest 7 is for the testing dataset. We employed STNN to predict the 7-step-ahead values for four different target variables, which are shown in Table S6.

Table S6. The prediction result of TF data.

Dataset	Metric	Methods						
		STNN	STNN*	ARIMA	SVR	RBF	RNN	KAE
y_1	PCC	0.996	0.888	0.994	**0.998**	0.981	**0.998**	0.985
	nRMSE	**0.150**	0.428	0.308	0.216	1.132	0.815	7.583
y_2	PCC	**1.000**	0.849	1.000	0.999	0.993	0.998	0.972
	nRMSE	**0.152**	1.058	0.102	0.407	2.106	0.721	4.720
y_3	PCC	0.962	0.751	0.297	0.951	**0.996**	-0.955	0.987
	nRMSE	**0.135**	0.719	0.858	0.624	1.083	1.354	8.317
y_4	PCC	**0.999**	0.896	0.993	**0.999**	0.989	0.986	-0.311
	nRMSE	**0.048**	0.941	0.069	0.272	1.129	0.320	6.552

4.7 Robustness

To test the robustness of STNN model, we increase the noise strength (σ) on pendulum data and explore the change of prediction accuracy. Fig.2 shows the change of PCC and NRMSE with noise strength (σ) from 0 to 0.5 on pendulum data for five different methods. The results are list in Table S7.

Table S7. The prediction result of robustness experiment.

Noise strength(σ)	Metric	Methods				
		STNN	SVR	RBF	RNN	KAE
0.00	PCC	0.995	0.991	0.993	0.971	0.990
	nRMSE	0.078	0.178	0.190	0.213	0.129
0.05	PCC	0.995	0.989	0.993	0.965	0.988
	nRMSE	0.081	0.178	0.187	0.213	0.144
0.10	PCC	0.994	0.986	0.992	0.976	0.986
	nRMSE	0.089	0.178	0.186	0.197	0.157
0.15	PCC	0.992	0.982	0.989	0.976	0.984
	nRMSE	0.100	0.178	0.186	0.192	0.203
0.20	PCC	0.990	0.977	0.987	0.972	0.979
	nRMSE	0.114	0.183	0.188	0.210	0.210
0.25	PCC	0.987	0.970	0.982	0.971	0.973
	nRMSE	0.130	0.204	0.197	0.215	0.239
0.30	PCC	0.984	0.965	0.977	0.970	0.969
	nRMSE	0.150	0.205	0.204	0.234	0.273
0.35	PCC	0.979	0.958	0.970	0.973	0.963
	nRMSE	0.170	0.226	0.216	0.237	0.280
0.40	PCC	0.975	0.950	0.962	0.962	0.957
	nRMSE	0.188	0.243	0.229	0.242	0.300
0.45	PCC	0.969	0.941	0.954	0.963	0.950
	nRMSE	0.209	0.262	0.242	0.252	0.319
0.50	PCC	0.964	0.930	0.946	0.956	0.944
	nRMSE	0.231	0.288	0.259	0.250	0.333

4.8 Dimension experiment

The dimension experiment results are shown in Fig.S4.2 and Table S8, and there are three approximate line level 1-2.

The line level 1 is $y = 5.67$, the line level 2 is $y = 5.83$ and the line level 3 is $y = 4.8M + 11.867$.

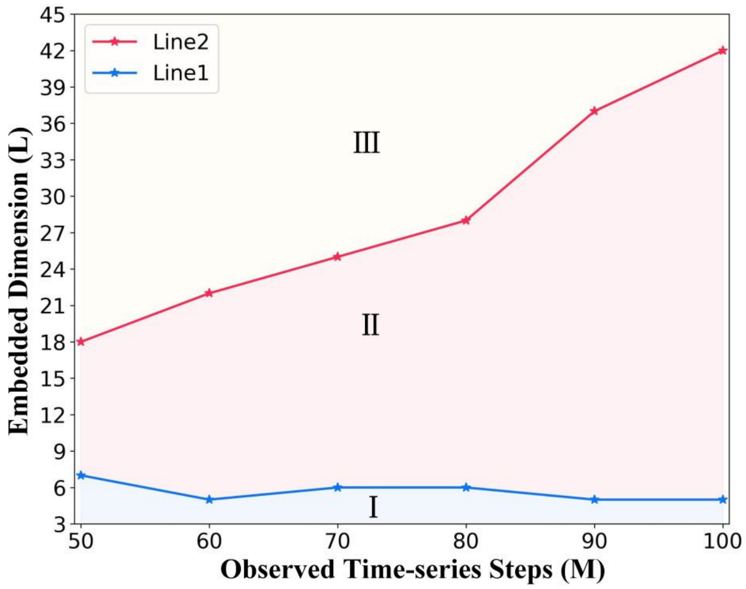

Figure S4.2. The change of the embedded dimension with the data length.

Table S8.1 The Forecasting metrics variation with embedded dimension L on observed time-series step on 50.

Embedding Dimension(L)	NRMSE				
	Dim1	Dim2	Dim3	Dim4	Mean
3	2.8828	3.2903	3.5746	0.7662	2.6285
4	0.4795	2.0223	4.8888	0.7721	2.0407
5	0.3152	0.5720	2.5662	0.3644	0.9545
6	0.6637	0.4173	1.3762	1.3913	0.9621
**7**	**1.5788**	**0.9155**	**0.4574**	**1.4405**	**1.0980**
8	0.4990	0.3998	0.1524	0.4924	0.3859
9	0.6054	0.4393	0.3908	0.5124	0.4870
10	1.3205	0.4625	0.4145	0.2400	0.6094
11	1.8008	0.9252	0.2334	0.2931	0.8131
12	1.7388	0.8588	0.1254	0.6587	0.8454
13	0.9662	1.4547	0.2035	0.3024	0.7317
14	0.2749	0.7654	0.0758	0.4828	0.3997
15	0.2575	0.5556	0.0611	0.3338	0.3020
16	0.2250	0.3115	0.2297	0.2911	0.2643
17	0.1827	0.3546	0.3128	0.2359	0.2715
18	0.2452	0.2626	0.2519	0.1411	0.2252
19	0.3042	0.1800	0.5359	0.2588	0.3197
20	0.5329	0.6450	0.3659	0.3707	0.4786
21	0.7572	1.2913	0.8516	0.6635	0.8909
22	0.4271	1.1849	1.0773	0.5840	0.8183
23	1.2413	1.6831	0.8743	0.7352	1.1335
24	1.7756	2.6136	0.3898	2.5998	1.8447
25	2.1650	1.7663	1.1050	1.4605	1.6242

Table S8.2 The Forecasting metrics variation with embedded dimension L on observed time-series step on 60.

Embedding Dimension(L)	NRMSE				
	Dim1	Dim2	Dim3	Dim4	Mean
3	1.1398	1.7951	4.1762	2.5055	2.4042
4	0.0621	0.0677	1.6360	1.5414	0.8268
**5**	**1.3993**	**0.4725**	**0.4425**	**0.5279**	**0.7105**
6	0.4670	1.4181	0.1704	0.2475	0.5758
7	0.5617	0.6400	0.3142	0.4410	0.4892
8	1.4021	0.4009	0.8396	0.4697	0.7780
9	1.5249	0.4163	0.9652	0.7988	0.9263
10	1.1014	0.4786	0.7142	0.5390	0.7083
11	0.7243	0.4496	0.7559	0.4477	0.5944
12	0.4503	0.0323	0.2998	0.4113	0.2984
13	0.3273	0.0611	0.0969	0.1452	0.1576
14	0.4618	0.0576	0.2233	0.1538	0.2241
15	0.2959	0.0400	0.0971	0.1349	0.1420
16	0.1612	0.0367	0.1406	0.1196	0.1145
17	0.1919	0.0219	0.1331	0.1716	0.1296
18	0.1538	0.0266	0.0977	0.1240	0.1005
19	0.1638	0.1434	0.1196	0.1320	0.1397
20	0.3090	0.0568	0.1493	0.1085	0.1559
21	0.3274	0.0421	0.2406	0.2425	0.2131
22	0.1999	0.1146	0.1880	0.1350	0.1593
23	0.1536	0.6463	0.4077	0.3399	0.3869
24	0.4026	0.4588	0.4674	0.1795	0.3771
25	0.8323	1.3748	0.9963	0.3355	0.8847
26	0.5711	0.8120	0.9169	0.7658	0.7664
27	1.2081	1.9044	1.5974	1.2663	1.4941
28	0.6795	0.8061	1.4137	1.5453	1.1111
29	1.0231	0.6893	1.1442	1.7078	1.1411
30	1.0206	0.9491	2.6420	2.8121	1.8560

Table S8.3 The Forecasting metrics variation with embedded dimension L on observed time-series step on 70.

Embedding Dimension(L)	NRMSE				
	Dim1	Dim2	Dim3	Dim4	Mean
3	18.1332	1.2142	2.0153	0.9152	5.5695
4	1.9333	0.5974	4.8282	0.7437	2.0256
5	2.1615	1.1932	4.2251	0.8718	2.1129
**6**	**2.4311**	**1.1735**	**1.8931**	**1.0868**	**1.6461**
7	1.9580	0.7362	0.6561	0.8150	1.0413
8	1.6712	0.3407	0.2987	0.4254	0.6840
9	1.4558	0.6047	0.2808	0.9783	0.8299
10	1.0780	0.3446	0.2426	0.3576	0.5057
11	1.2238	0.6819	0.2081	1.1337	0.8119
12	1.4871	0.3055	0.1954	0.3270	0.5787
13	0.9408	0.2336	0.1435	0.3237	0.4104
14	0.9009	0.2862	0.1421	0.2844	0.4034
15	1.4783	0.2225	0.1285	0.3651	0.5486
16	1.0102	0.1844	0.1406	0.2729	0.4020
17	0.8588	0.1421	0.1068	0.1958	0.3259
18	0.1952	0.1013	0.1046	0.1256	0.1317
19	0.4638	0.0422	0.0528	0.0718	0.1577
20	0.4658	0.1074	0.0846	0.2042	0.2155
21	0.7427	0.2506	0.1328	0.2061	0.3330
22	0.6325	0.1841	0.1345	0.2865	0.3094
23	1.0631	0.1963	0.1501	0.2485	0.4145
24	0.8319	0.2058	0.1219	0.1716	0.3328
25	0.9336	0.1540	0.1243	0.1635	0.3439
26	1.4993	0.1843	0.1821	0.2143	0.5200
27	1.6023	0.2168	1.0448	0.1312	0.7488
28	1.4085	0.5290	1.1417	0.6026	0.9204
29	1.3382	0.8124	1.1876	1.0024	1.0852
30	2.4835	1.1328	1.5156	1.6198	1.6879
31	3.7070	2.3180	2.4966	3.2998	2.9554
32	2.6886	2.2531	2.4450	2.5196	2.4766
33	1.5552	1.4972	1.5697	1.3290	1.4878
34	1.2196	1.1572	1.3953	0.8046	1.1442

35	2.0798	2.2327	2.3047	1.4503	2.0169

Table S8.4 The Forecasting metrics variation with embedded dimension L on observed time-series step on 80.

Embedding Dimension(L)	NRMSE				
	Dim1	Dim2	Dim3	Dim4	Mean
3	0.8163	2.6240	0.2443	2.0234	1.4270
4	0.1134	2.3056	0.5231	1.7355	1.1694
5	0.8321	1.4892	0.7630	0.6873	0.9429
**6**	**0.2414**	**0.0478**	**0.4788**	**2.9478**	**0.9290**
7	0.2664	0.3522	0.3420	0.4593	0.3550
8	0.5238	0.3130	1.0886	0.4045	0.5825
9	0.5913	0.4277	0.1912	0.3187	0.3822
10	0.8416	0.2239	0.1631	0.1267	0.3388
11	0.1731	0.3062	0.3903	0.3262	0.2990
12	0.2650	0.4301	0.1380	0.6023	0.3589
13	0.0771	0.3963	0.1758	0.2294	0.2197
14	0.2249	0.2246	0.2795	0.3573	0.2716
15	0.0493	0.1983	0.1308	0.3420	0.1801
16	0.1303	0.1770	0.1718	0.2708	0.1875
17	0.0669	0.1303	0.1020	0.1178	0.1043
18	0.0404	0.1173	0.0680	0.0853	0.0778
19	0.1371	0.1069	0.0622	0.1045	0.1027
20	0.0532	0.2370	0.1177	0.1739	0.1455
21	0.0886	0.2598	0.1528	0.1894	0.1727
22	0.0186	0.1542	0.0934	0.1264	0.0982
23	0.0861	0.1305	0.0792	0.1322	0.1070
24	0.0777	0.1257	0.0826	0.0862	0.0931
25	0.1354	0.1227	0.0813	0.2490	0.1471
26	0.0476	0.1268	0.0874	0.1456	0.1019
27	0.1753	0.1646	0.0668	0.1578	0.1411
28	0.1652	0.1986	0.1155	0.2297	0.1773
29	0.2292	0.4250	0.1363	0.3180	0.2771
30	0.1882	0.2896	0.1711	0.4053	0.2636
31	0.1212	0.2570	0.2712	0.1172	0.1917

32	0.1939	0.7894	0.5380	0.1577	0.4198
33	0.1718	0.7741	0.4831	0.1289	0.3895
34	0.1163	0.6754	0.2030	0.0595	0.2636
35	0.2877	0.8991	0.2759	0.3752	0.4595
36	1.8945	0.3437	0.3833	0.3826	0.7510
37	1.5620	0.6351	0.3028	0.5476	0.7619
38	0.4859	1.0004	0.5944	0.3561	0.6092
39	1.4802	1.6679	0.8633	2.2118	1.5558
40	2.0917	2.2227	1.5415	1.4687	1.8312

Table S8.5 The Forecasting metrics variation with embedded dimension L on observed time-series step on 90.

Embedding Dimension(L)	NRMSE				
	Dim1	Dim2	Dim3	Dim4	Mean
3	2.4461	1.1981	1.2395	1.7130	1.6492
4	1.3743	0.6413	0.3841	1.9084	1.0770
**5**	**0.0595**	**0.9962**	**2.0325**	**2.8431**	**1.4828**
6	0.3719	0.5555	0.2697	0.2612	0.3646
7	0.0415	0.6403	0.4281	0.4733	0.3958
8	0.3127	0.4136	1.0034	0.9306	0.6651
9	0.2923	0.4570	0.9962	1.1007	0.7116
10	0.1843	0.5472	0.7410	0.7975	0.5675
11	0.2377	0.3335	0.5071	0.4971	0.3938
12	0.3402	0.0391	0.4053	0.1948	0.2448
13	0.1598	0.0281	0.2265	0.2681	0.1706
14	0.1584	0.0373	0.2554	0.1716	0.1557
15	0.0805	0.0224	0.1262	0.0950	0.0810
16	0.0877	0.0406	0.1652	0.1690	0.1156
17	0.0661	0.0197	0.1849	0.1778	0.1121
18	0.0531	0.0451	0.0558	0.0887	0.0607
19	0.1015	0.0260	0.1332	0.0911	0.0880
20	0.2274	0.0276	0.2341	0.0659	0.1388
21	0.1318	0.0446	0.2548	0.1084	0.1349
22	0.1197	0.0313	0.1317	0.1368	0.1048
23	0.1189	0.0187	0.1271	0.1647	0.1074

24	0.0959	0.0277	0.1625	0.1877	0.1185
25	0.1006	0.0270	0.2796	0.3401	0.1868
26	0.0892	0.0380	0.2583	0.2589	0.1611
27	0.0977	0.0563	0.1475	0.1683	0.1174
28	0.1882	0.0602	0.1986	0.1358	0.1457
29	0.0914	0.0391	0.2194	0.1490	0.1247
30	0.1627	0.0327	0.1837	0.1811	0.1400
31	0.1135	0.0318	0.1773	0.1423	0.1162
32	0.1951	0.0529	0.2152	0.1582	0.1553
33	0.0969	0.0229	0.2128	0.1302	0.1157
34	0.0387	0.0713	0.1147	0.1119	0.0841
35	0.1146	0.0657	0.1647	0.1330	0.1195
36	0.0833	0.0895	0.1393	0.1179	0.1075
37	0.1006	0.0847	0.2095	0.1923	0.1468
38	0.2413	0.2303	0.4274	0.1993	0.2746
39	0.1294	0.6289	0.6871	0.5503	0.4989
40	1.1013	1.0247	0.7707	0.4263	0.8308
41	1.0756	1.9387	2.2709	1.0866	1.5930
42	1.7588	1.7765	1.4187	0.3316	1.3214
43	1.2608	1.0119	0.9061	0.1818	0.8402
44	0.3811	0.8203	1.2453	1.1311	0.8944
45	0.4676	0.7560	0.7190	0.6634	0.6515

Table S8.6 The Forecasting metrics variation with embedded dimension L on observed time-series step on 100.

Embedding Dimension(L)	NRMSE				
	Dim1	Dim2	Dim3	Dim4	Mean
3	2.1064	0.8661	0.5977	1.1533	1.1809
4	0.2344	0.5328	0.5151	0.8125	0.5237
**5**	**0.4866**	**0.3750**	**0.5371**	**0.7241**	**0.5307**
6	0.4473	0.5212	0.2254	0.3368	0.3827
7	0.1888	0.1520	0.2706	0.1736	0.1963
8	0.8999	0.1194	0.2533	0.4698	0.4356
9	0.3013	0.4144	0.1146	0.1457	0.2440
10	0.3987	0.1361	0.0620	0.2554	0.2131

11	0.1958	0.0864	0.1675	0.1042	0.1385
12	0.4461	0.0862	0.0290	0.0907	0.1630
13	0.0486	0.0334	0.0372	0.0352	0.0386
14	0.2343	0.0391	0.0488	0.0629	0.0963
15	0.1307	0.0226	0.0312	0.0339	0.0546
16	0.2663	0.0269	0.0288	0.0445	0.0916
17	0.0761	0.0247	0.0181	0.0407	0.0399
18	0.0241	0.0230	0.0276	0.0535	0.0320
19	0.0434	0.0214	0.0244	0.0286	0.0295
20	0.0521	0.0222	0.0132	0.0337	0.0303
21	0.0534	0.0126	0.0182	0.0169	0.0253
22	0.0213	0.0159	0.0133	0.0206	0.0178
23	0.0884	0.0179	0.0175	0.0253	0.0373
24	0.0489	0.0198	0.0232	0.0218	0.0284
25	0.0634	0.0229	0.0258	0.0368	0.0372
26	0.0631	0.0487	0.0245	0.0267	0.0408
27	0.1014	0.0392	0.0342	0.0649	0.0599
28	0.1765	0.0407	0.0264	0.0656	0.0773
29	0.1699	0.0292	0.0225	0.0298	0.0628
30	0.1352	0.0340	0.0379	0.0379	0.0612
31	0.1583	0.0318	0.0337	0.0546	0.0696
32	0.1932	0.0291	0.0232	0.0765	0.0805
33	0.1393	0.0261	0.0200	0.0496	0.0587
34	0.1033	0.0298	0.0227	0.0213	0.0443
35	0.1289	0.0256	0.0296	0.0659	0.0625
36	0.1462	0.0365	0.0258	0.0219	0.0576
37	0.1607	0.0273	0.0347	0.0753	0.0745
38	0.2227	0.0431	0.0318	0.1153	0.1032
39	0.2221	0.0458	0.0580	0.1153	0.1103
40	0.4303	0.2005	0.0315	0.2075	0.2175
41	0.2274	0.1905	0.0895	0.1576	0.1662
42	0.1597	0.1922	0.0469	0.0950	0.1234
43	0.2486	0.2144	0.2417	0.2797	0.2461
44	1.1895	0.7873	0.6234	0.7420	0.8355
45	2.4689	2.2236	1.2898	1.2632	1.8114
46	1.4748	1.6871	1.5471	1.3495	1.5146
47	0.4755	1.7138	1.7611	1.9210	1.4678

48	1.3341	2.6054	2.0328	1.8301	1.9506
49	2.1340	2.3915	0.7074	1.2104	1.6108
50	1.0394	0.9596	1.1599	1.9062	1.2663

Table S8.7. The prediction result of dimension experiment.

Embedding Dimension(L)	Data length					
	50	60	70	80	90	100
level1	7	5	6	6	5	5
level3	18	22	25	28	37	42

4.9 The perfomance of STNN

Table S9.1. Forecasting metric variation with the observed time-series steps(M) on pendulum data.

Var	Metric	Embedding Dimension(M)			
		50	60	70	80
1	PCC	0.909	0.9798	0.9972	0.9981
	NRMSE	0.5448	0.2675	0.0838	0.0532
2	PCC	0.8359	0.9609	0.996	0.991
	NRMSE	0.8386	0.2323	0.122	0.237

Table S9.2. Forecasting metric variation with the observed time-series steps(M) on solar data.

Var	Metric	Embedding Dimension(M)			
		375	400	425	450
1	PCC	0.2945	0.2901	0.9381	0.945
	NRMSE	1.2469	0.6968	0.5132	0.45
2	PCC	0.5852	0.9572	0.9924	0.9944
	NRMSE	1.8573	0.3772	0.1276	0.1082

Table S9.3. Forecasting metric variation with the observed time-series steps(M) on TS data.

Var	Metric	Embedding Dimension(M)			
		45	60	75	90
1	PCC	0.1479	0.9125	0.9185	0.948
	NRMSE	1.1953	0.5887	0.4552	0.35411
2	PCC	0.0122	0.6617	0.6062	0.9172
	NRMSE	2.1012	1.2036	0.9442	0.5444

Table S9.4. Forecasting metric variation with the dimension of time-series(D) on pendulum data.

Var	Metric	Embedding Dimension(M)		
		24	44	64
1	PCC	0.9959	0.9943	0.9981
	NRMSE	0.0648	0.0746	0.0532
2	PCC	0.9866	0.9839	0.991
	NRMSE	0.2141	0.1621	0.237

Table S9.5. Forecasting metric variation with the dimension of time-series(D) on solar data.

Var	Metric	Embedding Dimension(M)			
		21	31	41	51
1	PCC	0.9483	0.9392	0.955	0.945
	NRMSE	0.4016	0.4664	0.4043	0.45
2	PCC	0.9941	0.9944	0.9932	0.9944
	NRMSE	0.1113	0.1095	0.1192	0.1082

Table S9.6. Forecasting metric variation with the dimension of time-series(D) on TS data.

Var	Metric	Embedding Dimension(M)			
		57	107	157	207
1	PCC	0.9742	0.9385	0.9692	0.948
	NRMSE	0.2918	0.4661	0.35	0.3541
2	PCC	0.9095	0.9088	0.901	0.9172
	NRMSE	0.5733	0.5652	0.6057	0.5444

Table S9.7. Forecasting metric variation with the embedded dimension(L) on pendulum data.

Var	Metric	Embedding Dimension(M)							
		5	10	15	20	25	30	35	40
1	PCC	0.999	0.9197	0.9968	0.9981	0.9947	0.9934	0.9972	-0.5222
	NRMSE	0.8321	0.8416	0.0493	0.0532	0.1354	0.1882	0.2877	2.0917
2	PCC	0.9533	0.9797	0.9833	0.991	0.9901	0.977	0.711	-0.1251
	NRMSE	1.4892	0.2239	0.1983	0.237	0.1227	0.2896	0.8991	2.2227

Table S9.8. Forecasting metric variation with the embedded dimension(L) on solar data.

Var	Metric	Embedding Dimension(M)							
		25	50	75	100	125	150	175	200
1	PCC	inf	inf	0.1199	0.8159	0.9123	0.945	0.6932	0.509
	NRMSE	inf	0.6554	0.5582	0.7331	0.4094	0.45	0.8726	3.177
2	PCC	inf	inf	0.8855	0.9957	0.9908	0.9944	0.7665	0.7649
	NRMSE	inf	0.1722	0.097	0.1076	0.1574	0.1082	0.6926	1.1566

Table S9.9. Forecasting metric variation with the embedded dimension(L) on TS data.

Var	Metric	Embedding Dimension(M)							
		5	10	15	20	25	30	35	40
1	PCC	0.8504	0.9687	0.9201	0.948	0.8568	0.6937	0.5735	-0.0007
	NRMSE	1.0136	0.3515	0.6944	0.3541	0.4906	0.7429	0.8787	1.6075
2	PCC	-0.7726	0.8406	0.9503	0.9172	0.8861	0.9398	0.8619	0.2572
	NRMSE	1.6579	0.2756	0.3665	0.5444	0.6685	0.547	0.7425	1.0726

5. Supplementary Figure

Supplementary Figure S5.1 The workflow of each computational experiment

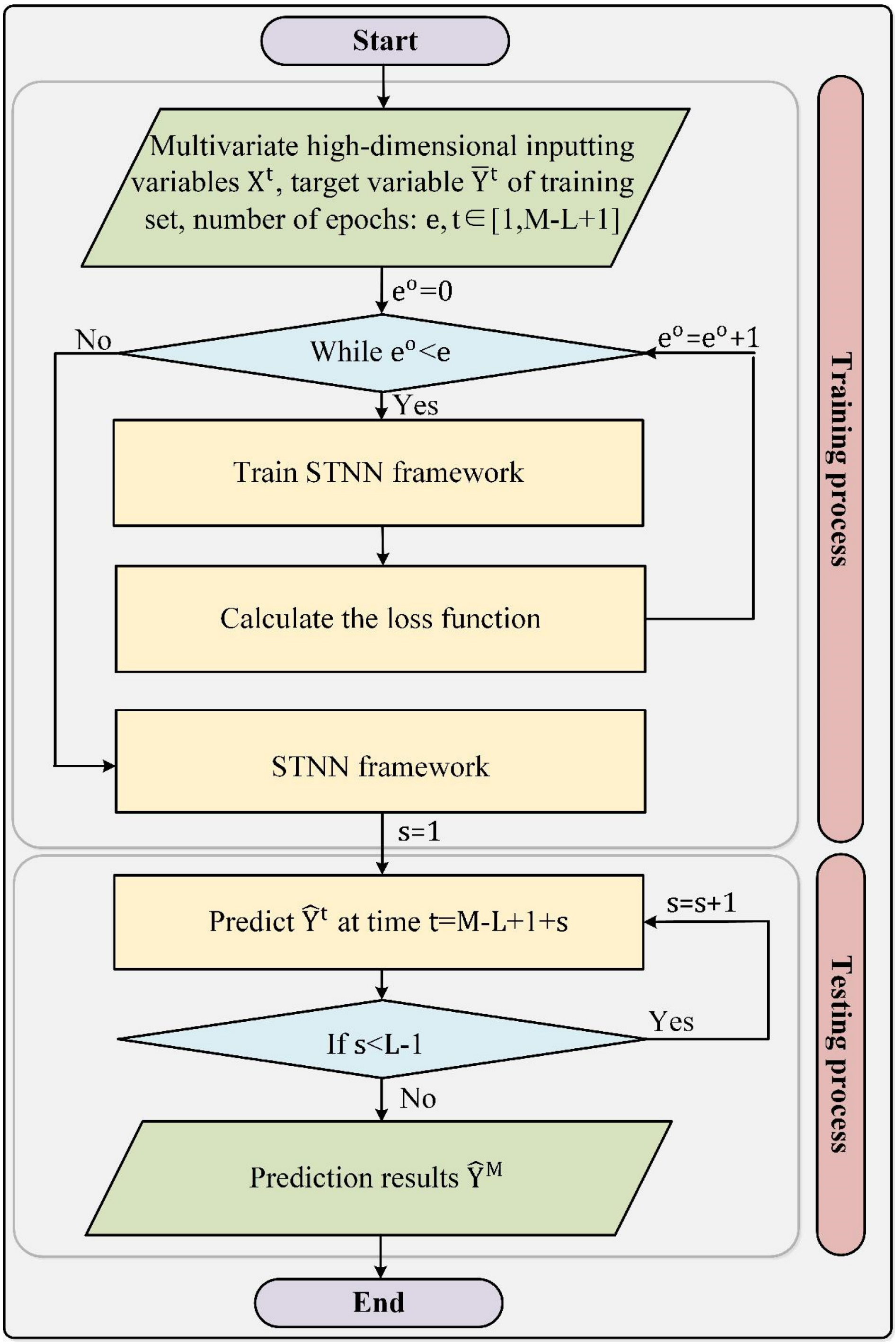

Figure S5.1. Workflow of each computational experiment.

6. Supplementary Algorithm

Supplementary Algorithm S1 The algorithm of each computational experiment

Algorithm 1: the workflow of each computational experiment

Input: Multivariate high-dimensional inputting variables X^t, target variable $\overline{Y}^t$ of training set

Parameter: Number of epochs: e, $t \in$ [1, M-L+1]

Output: Prediction results $\widehat{Y}^M$

1: Let $e^0 = 0$
2: **while** $e^0 < e$ **do**
3:     Train STNN framework
4:     Calculate the loss function
      $e^0 \leftarrow e^0 + 1$
5: **end while**
6: Obtain STNN framework
7: **Let** $s = 1$
8: Predict $\widehat{Y}^t$ at time t=M-L+1+s
9: **if** $s < L - 1$ **then**
10:     $s \leftarrow s + 1$; goto step 8
11: **end if**
12: **return** $\widehat{Y}^M$

7. **Supplementary codes**

The codes for STNN is available at https://github.com/347251369/STNN
